# Proportion of Adults Meeting the 2018 Physical Activity Guidelines for Americans According to Accelerometers

**DOI:** 10.3389/fpubh.2019.00135

**Published:** 2019-06-07

**Authors:** Zachary Zenko, Erik A. Willis, David A. White

**Affiliations:** ^1^Department of Kinesiology, California State University, Bakersfield, CA, United States; ^2^Center for Health Promotion and Disease Prevention, University of North Carolina-Chapel Hill, Chapel Hill, NC, United States; ^3^Children's Mercy Hospital, Ward Family Heart Center, Kansas City, MO, United States; ^4^School of Medicine, University of Missouri, Kansas City, MO, United States

**Keywords:** physical activity guidelines, physical activity behavior, accelerometers, physical activity surveillance, Physical Activity Guidelines for Americans

## Abstract

The new 2018 Physical Activity Guidelines for Americans provides updated recommendations for physical activity behavior. These guidelines remove the requirement for physical activity to be obtained in bouts of at least 10 min. The purpose of the present study was to provide an updated estimate of the proportion of adults meeting the physical activity guidelines, based on nationally representative data using accelerometers. Data from 6,525 adults were included in this study. The proportion of adults meeting the physical activity guidelines according to the Department of Health and Human Services and according to the American College of Sports Medicine were estimated using (a) lifestyle activities and (b) ambulatory activities only. Estimates of the proportion of adults meeting the physical activity guidelines ranged from 3.4 to 95.6%, even when based on the same data. The large range of these estimates suggest that challenges exist when using accelerometer data to estimate the levels of physical activity behavior in the population. Further, the large range indicates that, perhaps, physical activity guidelines should not be used as a reference point for behavior change. Instead, we suggest that efforts should be made to promote physical activity in reference to current behavior.

In 2018, the U.S Department of Health and Human Services (DHHS) and the Physical Activity Guidelines Advisory committee published the second edition of the Physical Activity Guidelines for Americans (1). Although much of the guidelines are consistent with the previous edition, the new physical activity guidelines indicate “moderate-to-vigorous physical activity (MVPA) of any duration may be included in the daily accumulated total volume of physical activity” (p. A-5), contrasting with the prior guidelines, which indicated that MVPA had to be in bouts of at least 10 min in duration.

According to the previous set of guidelines, estimates of physical activity behavior using device-based measurement (i.e., accelerometers) indicate that the percentage of adults achieving sufficient levels of physical activity is extremely low (2, 3). However, since removing the 10-min bout requirement, device-based estimates of physical activity prevalence according to the new guidelines have not been documented. With this change, it is likely that the proportion of adults meeting the physical activity guidelines will artificially change or be corrected, not based on any difference in population behavior, but due to the change in the physical activity guideline itself. If every minute of physical activity counts, then previous estimates of population levels of physical activity have likely been underestimated.

To quantify the prevalence of adults meeting the new guidelines, we estimated the proportion of the adult population meeting the physical activity guidelines, with and without the 10-min bout requirement. The criteria for meeting the physical activity guidelines were based on (a) the DHHS Physical Activity Guidelines for Americans (1), and (b) the American College of Sports Medicine (ACSM) (4). The DHHS and ACSM physical activity guidelines differ slightly because the DHHS Physical Activity Guidelines for Americans recommends 150 min of moderate-intensity equivalent activity per week, while the ACSM recommends a combination of days and minutes per week (30 min on at least 5 days per week) of moderate-intensity equivalent activity. Only the DHHS Physical Activity Guidelines for Americans removed the 10-min minimum bout requirement. We decided to also use the ACSM guideline to estimate the proportion of the adult population meeting the physical activity recommendation because we speculate that the ACSM may follow the lead of the DHHS and also remove the 10-min minimum bout requirement when their guidelines are eventually updated. Thus, we aimed to be more informative and complete in our analyses.

In addition, for each set of guidelines, we estimated the proportion of adults meeting the physical activity guidelines when MVPA was defined by “lifestyle activities” (accelerometer count threshold representing activities of daily living) and “ambulatory activities” (accelerometer count threshold representing exercise specifically related to walking/running activities). Together, these data describe several estimates of physical activity prevalence that are based on (a) DHHS 2018 and ACSM's 2011 physical activity guidelines for Americans with and without the 10-min minimum bout requirement, and (b) two cut-point criteria for defining MVPA. Our analyses were based on accelerometer data, thus limiting our analyses to the proportion of adults meeting the cardiorespiratory, “aerobic” guidelines only.

## Methods

We analyzed data from the 2003–2004 and 2005–2006 cycles of the National Health and Nutrition Examination Survey (NHANES), of U.S. children and adults (5), which uses a stratified multistage probability sampling design to produce a nationally representative sample of the civilian non-institutionalized U.S. population. More recent datasets are not yet available. We limited our potential study sample to the 10,637 NHANES respondents aged 18 years or older who participated in both the interview and physical examination components of the survey. Of 10,637 adults who participated in both the interview and examination components of NHANES during 2003–2006, accelerometer data was collected on 9,601 of the participants. Of these we excluded data from 3,076 participants for the following reasons: accelerometer not calibrated or reliable, <4 valid days, or missing demographic or anthropometric data. The analytic cohort included 6,525 adults (3,305 women and 3,320 men; Table 1). The Centers for Disease Control and Prevention Ethics Review Board approved the survey protocols, and all adults who participated in the survey provided their informed consent.

**Table 1:** Distribution of characteristics among study participants, National Health and Nutrition Examination Survey (NHANES) 2003–2006 (*n* = 6,525).

	**Unweighted**		**Weighted**	
	***n***	**%**	**%**	**(95% CI)**
**Sex**				
Male	3,220	49.4	48.1	(46.7, 49.6)
Female	3,305	50.7	51.9	(50.4, 53.3)
**Age group**				
18–24 years	876	13.4	11.2	(9.9, 12.5)
25–44 years	1,991	30.5	39.2	(36.5, 41.8)
45–64 years	1,926	29.5	33.0	(30.6, 35.4)
65+ years	1,926	29.5	16.6	(15.0, 18.3)
**Race/Ethnicity**				
Non-Hispanic White/other	3,600	55.2	77.0	(72.8, 81.2)
Non-Hispanic Black	1,329	20.4	11.5	(8.6, 14.5)
Hispanic	1,596	24.5	11.5	(8.8, 14.2)
**Body Mass Index (BMI; kg/m**^**2**^**)**				
Underweight	104	1.6	1.7	(1.4, 2.0)
Healthy weight	2,042	31.3	32.8	(30.9, 34.8)
Overweight	2,042	31.3	34.0	(32.0, 35.9)
Obese	2,082	31.9	31.5	(29.3, 33.7)

*CI, confidence interval. Underweight, normal, overweight, and obese classifications are on the basis of body mass index, which is weight (kg)/height (m)^2^. Underweight, <18.5; healthy, 18.5 24.9; overweight, 25.0–29.9; and obese, ≥30.0*.

### Measures

The NHANES participants were classified by sex, age (18–24, 25–44, 45–64, or ≥65 years), race/ethnicity (non-Hispanic White/other, non-Hispanic Black, or Hispanic), and body mass index (BMI; kg/m^2^) category (underweight: <18.5 kg/m^2^, healthy weight: 18.5–25.0 kg/m^2^, overweight: 25.0–29.9 kg/m^2^, obese: ≥30 kg/m^2^).

Physical activity was assessed with a uniaxial accelerometer (ActiGraph model 7164, LLC, Ft. Walton Beach, FL) that participants wore for 7 days over their right hip on an elasticized belt except when they were sleeping or in contact with water (such as when bathing or swimming) (2). Accelerometer data were summed and scored at 60 s epochs. At the end of the 7-day activity assessment period, participants returned their accelerometers by mail, where NHANES personnel downloaded the data and checked to determine whether the calibration of the accelerometer was still within manufacturer's specifications.

Raw accelerometer counts were processed using the National Cancer Institute's statistical SAS programming code for aggregating data from the accelerometer (http://riskfactor.cancer.gov/tools/nhanes_pam/). The analysis determined how long participants wore their accelerometer on each of the 7 days and to estimate the number of minutes they engaged in bouted (8–10 min) and non-bouted (1-min) physical activity of both moderate- and vigorous-intensity. A valid day was determined as 10 or more hours of wear time. Non-wear time was assessed as any time interval with 60 or more minutes of continuous zero counts, allowing for a 1- to 2-min interruption with counts between 0 and 100 (2).

### Data Processing

MVPA equivalent activity was calculated as the sum of time spent in moderate-intensity activity plus twice the time spent in vigorous-intensity activity (6). Unfortunately, only 26% of the sample had seven valid days of wear time. Thus, guideline-specific analysis strategies were used to maximize the sample size to estimate adherence prevalence. The data processing and imputation approach described by Watson et al. (6) was followed to estimate the proportion of adults meeting the DHHS Physical Activity Guidelines for Americans (with and without setting a minimum bout duration of 10 min). The Bayesian approach described by Troiano et al. (2) was followed to estimate the proportion of adults meeting the physical activity guidelines according to the ACSM (with and without setting a minimum bout duration of 10 min). This approach focused on the probability of exercising for 30 min per day on at least 5 days per week, which is in accordance with the ACSM guidelines. These differing approaches were necessary because the DHHS Physical Activity Guidelines for Americans sets an equivalent of 150 min of MVPA per week, total, whereas the ACSM's guidelines includes a combination of days and minutes of moderate-intensity physical activity for each week.

We further estimated the proportion of adults meeting either physical activity guideline using MVPA based on two cut points: (1) “lifestyle activity,” based on the 760 cut point using estimated MET expenditure on the basis of other common physical activities that people engage in addition to walking and running (e.g., gardening, raking, mowing, vacuuming, sweeping, mopping, playing with children, and loading/unloading boxes) (7–10); and (2) “ambulatory activity,” based on the 2020 cut point using validation studies that estimated MET expenditure solely on the basis of walking and running (2).

### Statistical Analyses

We estimated the physical activity prevalence for the overall sample and by the following demographic and anthropometric characteristics: age group, sex, race/ethnicity, and BMI category. In all analyses, we used SURVEYFREQ and SURVEYMEANS procedures implemented in SAS version 9.4 (Research Triangle Institute, Research Triangle Park, NC) to account for the stratification, clustering, and weighting used in the complex survey design. Adjusted sample weights for subsamples with four or more valid days were used for all analyses (http://riskfactor.cancer.gov/tools/nhanes_pam/).

## Results

### Estimates According to the DHHS Physical Activity Guidelines for Adults

When the U.S. DHHS 2018 Physical Activity Guidelines for Americans and the criterion for MVPA using the cut point for “ambulatory” activities were applied, the proportion of sufficiently active adults was 9.7 ± 0.7% when a 10-min bout was required, and 44.8 ± 1.3% when a 10-min bout was not required (counting every minute). When the criterion for MVPA using the cut point for “lifestyle” activities was applied, these proportions increased to 57.9 ± 0.9% and 95.6 ± 0.3%, respectively. Proportions of sufficiently active adults based on subgroup analyses (i.e., BMI, age groups, sex, and ethnicity) are presented in Table 2.

**Table 2:** Prevalence^*^ (% and SEM) of the population attainting sufficient^#^,^†^ physical activity to meet public health recommendations.

	**Lifestyle activities**		**Ambulatory activities**	
	**Counting only bouts^**a**^**	**Counting every minute^**a**^**	**Counting only bouts^**b**^**	**Counting every minute^**b**^**
**A: 30+** **minutes 5 days/week**^**#**^				
**All adults**	57.5 (0.8)	77.6 (0.5)	3.4 (0.2)	15.0 (0.6)
**BMI (kg/m**^**2**^**)**				
18.5–25.0	59.9 (1.1)	79.1 (0.6)	4.6 (0.4)	18.6 (0.8)
25.0–30.0	59.0 (1.2)	78.2 (0.7)	4.0 (0.4)	16.5 (1.0)
30.0+	53.7 (1.2)	75.5 (0.9)	1.6 0.3	9.4 (0.7)
**Age groups**				
18–25 years	64.3 (1.2)	83.4 (0.7)	6.2 (0.9)	23.1 (1.5)
25–45 years	69.0 (1.0)	85.9 (0.4)	4.6 (0.3)	19.1 (0.9)
45–64 years	59.1 (1.3)	80.9 (0.7)	2.5 (0.3)	13.0 (0.6)
65+ years	23.0 (1.2)	47.7 (1.4)	0.7 (0.2)	3.7 (0.5)
**Sex**				
Male	63.4 (0.9)	80.0 (0.5)	5.7 (0.4)	21.9 (0.9)
Female	52.1 (1.0)	75.4 (0.7)	1.3 (0.2)	8.6 (0.5)
**Race/Ethnicity**				
Non-Hispanic White/other	56.6 (1.0)	77.1 (0.6)	3.0 (0.3)	14.3 (0.6)
Non-Hispanic Black	55.9 (1.1)	76.2 (0.8)	3.3 (0.3)	13.4 (1.1)
Hispanic	65.7 (0.9)	82.6 (0.6)	6.7 (0.5)	21.1 (1.2)
**B: 150+** **minutes/week**^†^				
**All adults**	57.9 (0.9)	95.6 (0.3)	9.7 (0.7)	44.8 (1.3)
**BMI (kg/m**^**2**^**)**				
18.5–25.0	63.0 (1.3)	96.1 (0.4)	14.2 (1.1)	53.4 (1.7)
25.0–30.0	61.4 (1.7)	95.9 (0.4)	10.4 (1.1)	47.7 (1.7)
30.0+	48.7 (1.8)	94.9 (0.5)	4.2 (0.4)	33.0 (1.8)
**Age groups**				
18–25 years	65.6 (2.1)	98.9 (0.6)	13.6 (1.7)	63.2 (2.6)
25–45 years	67.6 (1.3)	99.9 (0.0)	10.9 (1.2)	57.9 (1.7)
45–64 years	57.9 (1.2)	97.8 (0.3)	8.8 (0.8)	39.6 (1.6)
65+ years	29.8 (1.7)	78.9 (0.9)	6.0 (0.8)	12.3 (1.1)
**Sex**				
Male	68.2 (0.9)	97.0 (0.3)	11.3 (0.9)	58.1 (1.4)
Female	48.3 (1.2)	94.3 (0.4)	8.2 (0.7)	32.6 (1.4)
**Race/Ethnicity**				
Non-Hispanic White/other	56.9 (1.1)	95.1 (0.3)	9.5 (0.7)	43.9 (1.4)
Non-Hispanic Black	54.0 (1.8)	95.9 (0.6)	8.9 (1.1)	41.5 (2.0)
Hispanic	68.1 (1.5)	98.5 (0.3)	11.8 (1.2)	54.8 (2.1)

* *Prevalence estimates were based on individuals with four or more valid days of accelerometer data*.

a *Adherence definitions were based moderate-intensity criterion = 760 counts per minute*.

b *Adherence definitions were based moderate-intensity criterion = 2,020 counts per minute*.

# *Adherence, 30 min of moderate-intensity equivalent minutes of activity on 5 of 7 days, accumulating every minute above criterion and in modified 10 min bouts (8 of 10 min)*.

† *Adherence, ≥150 min of moderate-intensity equivalent minutes of activity per week, accumulating every minute above criterion and in modified 10-min bouts (8 of 10 min)*.

### Estimates According to the ACSM Physical Activity Guidelines for Adults

According to the ACSM's 2011 physical activity guidelines and the criterion for MVPA using the cut point for “ambulatory” activities, the proportion of sufficiently active adults was 3.4 ± 0.2% when a 10-min bout was required, and 15.0 ± 0.6% when a 10-min bout was not required (counting every minute). When the criterion for MVPA using the cut point for “lifestyle” activities, these proportions increased to 57.5 ± 0.8 and 77.6 ± 0.5%, respectively. Proportions of sufficiently active adults based on subgroup analyses (i.e., BMI, age groups, sex, and ethnicity) are presented in Table 2.

## Discussion

The purpose of this study was to report proportion of adults meeting the DHHS 2018 Physical Activity Guidelines for Americans and the ACSM's 2011 physical activity guidelines; comparing the previous guidelines, requiring 10-min bouts of continuous movement, and the recent change that removed the 10-min bout requirement. We further estimated these proportions using cut points representing “lifestyle” activities and “ambulatory” activities. When considering all adults combined and both cut points, regardless of BMI, age group, sex, and ethnicity, estimates of the proportion of sufficiently active adults ranged from 3.4 to 95.6%.

### Subgroup Analyses

In terms of lifestyle activities, people with a normal weight status tended to be more physically active than people with overweight or obesity, regardless of whether the activity was contingent on the 10-min bout requirement or counted every minute. Likewise, males were more active than females, and physical activity tended to decrease in the age groups older than 45 years. When considering ambulatory activities, people with a normal weight status tended to be more physically active than people with overweight or obesity. Similarly, physical activity tended to decrease with age, and males tended to be more physically active than females. Regardless of DHHS or ACSM guideline, activity type (lifestyle or ambulatory), and 10-min bout requirement, Hispanics were more active than Non-Hispanic Whites and Non-Hispanic Blacks. This indicates that Non-Hispanic Whites and Non-Hispanic Blacks may require more targeted physical activity promotion and intervention. Overall, these data indicate that some disparities (e.g., males being more active than females, adults with normal weight being more active than adults with obesity) are consistent regardless of the method used for estimation or the definition of the physical activity guidelines, and consistent with those disparities previously reported (2, 6).

### Implications for Public Health Promotion

When considering lifestyle activities, the proportion of adults meeting the 2018 DHHS Physical Activity Guidelines for Americans is 95.6%. This is considerably more optimistic than estimates based on ambulatory activities only and use of the 2020 counts per minute moderate-intensity criterion, which suggest 44.8% of adults meet the 2018 DHHS Physical Activity Guidelines. In turn, suggesting the proportion of sufficiently active adults is 44.8% is still more considerably more optimistic than previously indicated based on the ACSM guidelines, counting only bouts of physical activity (2, 3).

The consideration of different cut points (e.g., 760 counts per minute vs. 2020 counts per minute) has important implications for public health promotion. Consideration of the lifestyle activities in this study may overestimate MVPA because of the inclusion of light-intensity physical activity; on the other hand, restriction to only higher intensity MVPA in the 2020 counts per minute criterion may increase error due to the inability to track non-ambulatory activities, such as bicycling or gardening (9). Thus, the range of the proportion of adults meeting the 2018 DHHS Physical Activity Guidelines is large (50.8%), and dependent on cut-point criterion (see Table 2). The “true” proportion of adults meeting the 2018 DHHS Physical Activity Guidelines is likely between 44.8 and 95.6% [also see (11)].

It is possible that the potential inclusion of light-intensity activity is not a severe issue. Although moderate- and vigorous-intensity activity appear to be associated with greater reductions in mortality than light-intensity activity, light-intensity activity still has benefits (9). Further, mounting evidence and previous reviews indicate that substantial benefits can be obtained with levels of activity that are <150 min per week of MVPA (12). Indeed, perhaps the general message of “move more and sit less” that is presented throughout the 2018 DHHS Guidelines should be the focus, not whether adults are actually meeting the 2018 DHHS Physical Activity Guidelines themselves.

Does this wide range in prevalence of meeting the physical activity guidelines (45 to 96%) suggest that physical activity behavior only needs to increase in the least active of the population (55 or 4%)? We do not have any illusions that nearly half or nearly all adults are sufficiently active, or that the “pandemic” of physical activity was solved because the 10-min bout of continuous MVPA requirement was removed (13, 14). Together, our findings suggest that perhaps health- and physical activity- promotion efforts and definitions of success should not be in reference to the Physical Activity Guidelines. Instead, we contend that efforts should be focused on increasing physical activity in general. Further, we contend that any physical activity is beneficial—including lifestyle activities. We do not see compelling reason to restrict “meaningful” physical activities to ambulatory activities alone. Perhaps, then, people interested in promoting physical activity behavior and public health should focus on more holistic approaches and more broad definitions of success, rather than simply increasing the proportion of adults meeting the Physical Activity Guidelines for Americans based on accelerometer data.

### Implication for Public Health Surveillance

There are a few factors that should be considered when assessing population level physical activity in the current analysis and in the future. First, the data used in this study, and other studies using NHANES waist worn accelerometers, are more than 12 years old and may not be representative of the current population of U.S. adults. Second, in regard to longitudinal surveillance, findings in the present analysis found that the estimates of adults who are considered sufficiently active will be significantly higher with adoption of new physical activity guidelines. Simultaneously with the update of the guidelines, public health surveillance studies such as NHANES has shifted to wrist-worn rather than waist worn accelerometers. Although wrist worn accelerometry has several notable advantages (15), this increases the variability in the physical activity data and requires different cut points representing MVPA for both “lifestyle” and “ambulatory” activities, adding to the “intensity cut point conundrum” highlighted by Trost and colleagues (16, 17). Together, these changes present significant inconsistencies which will increase the difficulty of longitudinal tracking of physical activity behavior in the United States. However, with rapidly advancing research, expanding our understanding of physical activity behaviors, and effects on cardiometabolic health; as well as advances in methodology for objectively assessing physical activity, it would be unwise for public health guidelines and surveillance methodology to remain stagnant. This dilemma will present future physical activity epidemiologists with unique challenges that have yet to be solved.

Together, these data suggest that we must clarify (a) the problem of physical inactivity behavior, (b) the criteria for meaningful increases in physical activity behavior, and (c) strategies for accurate longitudinal physical activity guideline surveillance. We suggest that there is still more physical activity to promote and a need for behavior-change interventions, even using the most optimistic estimates of physical activity behavior, and that perhaps it is time to reconceptualize what is an active lifestyle, and if it depends on meeting the physical activity guidelines.

## Data Availability

Publicly available datasets were analyzed in this study. This data can be found here: http://www.cdc.gov/nchs/about/major/nhanes/datalink.htm.

## Ethics Statement

The Centers for Disease Control and Prevention Ethics Review Board approved the survey protocols, and all adults who participated in the survey provided their informed consent.

## Author Contributions

ZZ conceptualized the study and organized the development of the manuscript. EW organized and analyzed the data and gave critical contributions to the manuscript. DW gave critical feedback and contributions to the manuscript.

## Contributions to the Field

This study provides an updated estimate of the proportion of adults meeting the 2018 Physical Activity Guidelines for Americans, based on nationally representative data using accelerometers. Using a variety of guidelines and cut-points, we show that estimates of the adults meeting the physical activity guidelines vary widely, from 3.4 to 95.6% of the adult population. We suggest that the (a) problem of physical activity behavior, (b) the criteria for meaningful increases in physical activity behavior, and (c) strategies for accurate longitudinal physical activity guideline surveillance must be clarified.

### Conflict of Interest Statement

The authors declare that the research was conducted in the absence of any commercial or financial relationships that could be construed as a potential conflict of interest.

## References

[1] Physical Activity Guidelines Advisory Committee 2018 Physical Activity Guidelines Advisory Committee Scientific Report. Washington, DC: U.S. Department of Health and Human Services (2018).

[2] Troiano RP Berrigan D Dodd KW Mâsse L Tilert T McDowell M. Physical activity in the United States measured by accelerometer. Med Sci Sports Exerc. (2008) 40:181–8. 10.1249/mss.0b013e31815a51b3 18091006

[3] Tudor-Locke C Brashear MM Johnson WD Katzmarzyk PT. Accelerometer profiles of physical activity and inactivity in normal weight, overweight, and obese U.S. men and women. Int J Behav Nutr Phys Act. (2010) 7:60. 10.1186/1479-5868-7-60 20682057 PMC2924256

[4] Garber CE Blissmer B Deschenes MR Franklin BA Lamonte MJ Lee IM. Quantity and quality of exercise for developing and maintaining cardiorespiratory, musculoskeletal, and neuromotor fitness in apparently healthy adults: guidance for prescribing exercise. Med Sci Sports Exerc. (2011) 43:1334–59. 10.1249/MSS.0b013e318213fefb 21694556

[5] Centers for Disease Control and Prevention (CDC) National Center for Health Statistics (NCHS). National Health and Nutrition Examination Survey Data. Hyattsville, MD: U.S. Department of Health and Human Services, Centers for Disease Control and Prevention, (2006). Available online at: http://www.cdc.gov/nchs/about/major/nhanes/datalink.htm

[6] Watson KB Carlson S Carroll DD Fulton J Comparison of accelerometer cut points to estimate physical activity in U.S. adults. J Sports Sci. (2014) 32:660–9. 10.1080/02640414.2013.847278 24188163 PMC4589136

[7] Crouter SE DellaValle DM Haas JD Frongillo EA Bassett DR. Validity of the ActiGraph 2-regression model, Matthews cut-points, and NHANES cut-points for assessing free-living physical activity. J Phys Act Health. (2013) 10:504–14. 10.1123/jpah.10.4.504 22975460 PMC4199088

[8] Matthew CE. Calibration of accelerometer output for adults. Med Sci Sports Exerc. (2005) 37:S512–22. 10.1249/01.mss.0000185659.11982.3d 16294114

[9] Saint-Maurice PF Troiano RP Berrigan D Kraus WE Matthews CE Volume of light versus moderate-to-vigorous physical activity: similar benefits for all-cause mortality? J Am Heart Assoc. (2018) 3:e008815 10.1161/JAHA.118.008815 PMC5907606 29610219

[10] Welk GJ McClain JJ Eisenmann JC Wickel EE. Field validation of the MTI actigraph and bodymedia armband monitor using the IDEEA monitor. Obesity. (2007) 15:918–28. 10.1038/oby.2007.624 17426327

[11] Migueles JH Cadenas-Sanchez C Tudor-Locke C Löf M Esteban-Cornejo I Molina-Garcia P. Comparability of published cut-points for the assessment of physical activity: implications for data harmonization. Scand J Med Sci Sports. (2018) 29:566–74. 10.1111/sms.13356 30548545

[12] Warburton DER Bredin SSD. Reflections on physical activity and health: what should we recommend? Can J Cardiol. (2016) 32:495–504. 10.1016/j.cjca.2016.01.024 26995692

[13] Kohl HW Craig CL Lambert EV Inoue S Alkandari JR Leetongin G. The pandemic of physical inactivity: global action for public health. Lancet. (2012) 380:294–305. 10.1016/S0140-6736(12)60898-8 22818941

[14] Lee I-M Shiroma EJ Lobelo F Puska P Blair SN Katzmarzyk PT. Effect of physical inactivity on major non-communicable diseases worldwide: an analysis of burden of disease and life expectancy. Lancet. (2012) 380:219–29. 10.1016/S0140-6736(12)61031-9 22818936 PMC3645500

[15] Troiano RP McClain JJ Brychta RJ Chen KY. Evolution of accelerometer methods for physical activity research. Br J Sports Med. (2014) 48:1019–23. 10.1136/bjsports-2014-093546 24782483 PMC4141534

[16] Trost SG State of the art reviews: measurement of physical activity in children and adolescents. Am J Lifestyle Med. (2007) 1:299–314. 10.1177/1559827607301686

[17] Trost SG O'Neil M. Clinical use of objective measures of physical activity. Br J Sports Med. (2014) 48:178–81. 10.1136/bjsports-2013-093173 24311601

